# High aneurysm wall enhancement values are associated with late sac shrinkage after endovascular repair of abdominal aortic aneurysms

**DOI:** 10.1097/MD.0000000000024133

**Published:** 2021-01-15

**Authors:** Shinya Okata, Katsuyuki Hoshina, Kazuhiro Miyahara, Mitsuru Matsukura, Takafumi Akai, Toshihiko Isaji, Toshio Takayama

**Affiliations:** Division of Vascular Surgery, Department of Surgery, Graduate School of Medicine, The University of Tokyo, Tokyo, Japan.

**Keywords:** abdominal aortic aneurysm, aneurysm wall enhancement, sac, shrinkage

## Abstract

To analyze the correlation between aneurysm wall enhancement (AWE) values and early and late sac shrinkage after endovascular aneurysm repair (EVAR).

We retrospectively analyzed 28 patients who underwent EVAR for abdominal aortic aneurysms (AAA) using a bifurcated main body stent graft. The value of AWE in the slice of the maximum AAA diameter was measured using a volumetric analysis of computed tomography images. Sac measurements before EVAR and more than 10 months after EVAR were compared, and the maximum sac shrinkage rate was calculated.

The AWE value immediately after (4 to 7 days) EVAR correlated positively with the sac shrinkage rate (R^2^ = 0.0139). The AWE value at 6 months after EVAR was also strongly correlated with the sac shrinkage rate (R^2^ = 0.4982).

Higher AWE values at 6 months after EVAR were strongly associated with the sac volume shrinkage rate. High AWE values may be a predictive factor for sac shrinkage and may aid in the selection of the appropriate clinical strategy after EVAR.

## 1 Introduction

Endovascular aneurysm repair (EVAR) for abdominal aortic aneurysm (AAA) has been widely adopted worldwide in recent decades.^[1,2]^ Although numerous large population studies have demonstrated excellent short-term to mid-term outcomes,^[2–4]^ sac enlargement has remained one of the most critical long-term adverse events.^[5]^ Hoshina et al analyzed 38,003 EVAR patients and found that one-quarter of all AAA sacs had enlarged by more than 5 mm compared to their original diameters at 5 years postoperatively.^[6]^ This unexpectedly high rate of late enlargement has had a significant impact on the practices used by vascular surgeons and appears to have influenced operative indications, treatment strategies, and treatments after EVAR, including medication and catheter interventions.^[7–9]^

In an effort to predict future sac enlargement, Ito et al. reported that aneurysm wall enhancement (AWE) on contrast computed tomography (CT) could be a good predictor of sac shrinkage.^[10]^ They initially hypothesized that high AWE reflected inflammation and should predict sac expansion; however, the results ultimately showed the opposite, and the underlying mechanisms remain unknown. Their results were particularly interesting and encouraged us to introduce AWE measurements in our clinical practice. We aimed to analyze the correlation between AWE values and early (immediately [4–7 days] after EVAR) and late (6 months after EVAR) sac shrinkage after EVAR.

## 2 Materials and methods

### 2.1 Patients

A retrospective review of patients who underwent EVAR using bifurcated main body Excluder stent grafts (W.L. Gore & Associates, Flagstaff, AZ), Endurant stent grafts (Medtronic, Minneapolis, MN), or AFX stent- grafts (Endologix, Irvine, CA) from September 2009 to November 2018 was performed. All patients included in this study underwent preoperative and postoperative CT using a 64-detector row scanner. Contrast-enhanced CT was performed 120 second after intravenous administration of 600 mg/kg contrast agent over the course of 30 second. Patients who did not undergo contrast-enhanced CT and/or those with a follow-up duration less than 6 months after EVAR were excluded. The use of imaging data for this study was approved by the Ethics Committee of our institution [approval no. 3316-(3), 3252-(5)].

### 2.2 Measurements

We modified the methodology of a previous study^[10]^ to definitively confirm the usefulness of AWE. The previous study defined AWE as present when there was an increase of >20 Hounsfield units (HU) in the mean CT values at 1 month after EVAR. In our practice, we evaluated the AWE value using a volumetric analysis of CT images at 2 time points. The previous study defined expansion and shrinkage as a change of >5 mm in the maximal aneurysm diameter; however, we measured the sac volume itself to determine the rate of change.

**Figure 1:**
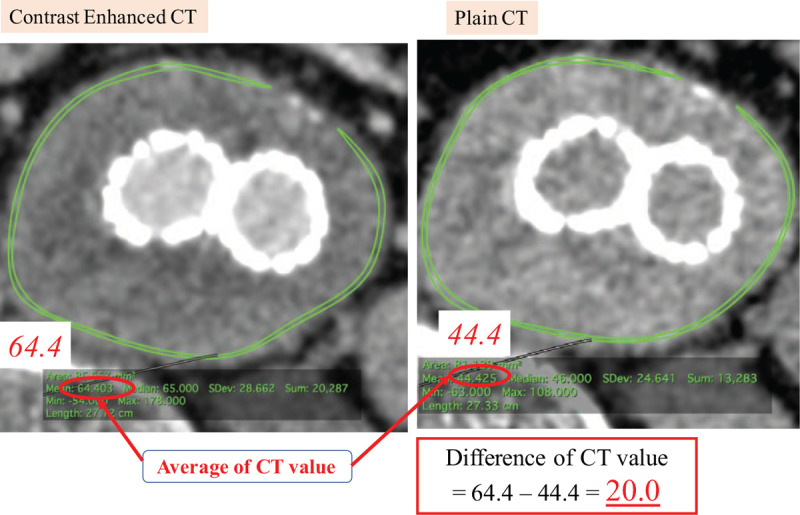
Definition of aortic wall enhancement. CT = computed tomography.

The CT images were transferred to a three-dimensional image analysis workstation (Osirix MD, v. 11.0.2) to measure the CT value and aneurysm sac volume. We selected 1 slice of the axial transverse view image that showed the maximal aneurysm diameter after EVAR. We manually circumscribed the aneurysm wall, excluding the calcified lesions, and the average CT value (HU) of the region of interest (ROI) was automatically calculated with Osirix software. We calculated the difference in the CT values of the contrast-enhanced images and plain images to determine the AWE value (Fig. 1). We also measured the preoperative and postoperative volume of the AAA sac by setting the ROI using Osirix. The extent of the change in the AAA sac was adjusted by comparing the axial CT images before and after EVAR. We defined the maximum change in the sac volume as the sac shrinkage rate.

### 2.3 Study design

**Figure 2:**
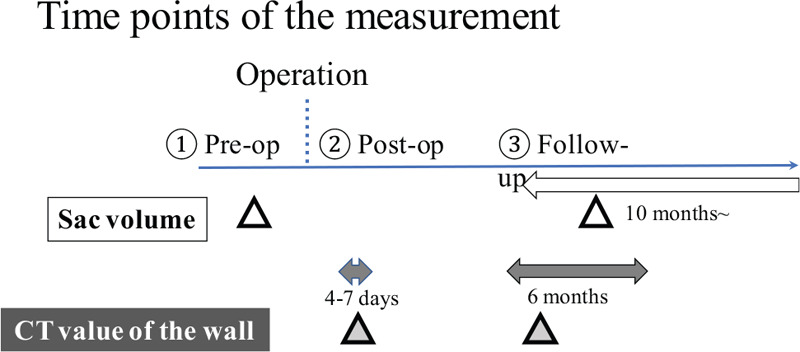
Measurement time points. CT = computed tomography.

The time points when measurements were performed are shown in Figure 2. The sac volumes were measured preoperatively and at least 10 months after EVAR (median, 22 months; range, 10–52 months). The AWE value after EVAR was calculated immediately (4–7 days) after EVAR and 6 months after EVAR (Fig. 2). For all measurements, we used the imaging data of the contrast-enhanced CT. To assess AWE and sac volumes, 2 of the authors (with 9 and 26 years of experience, respectively, interpreting vascular CT images) independently compared the images. The results of the 2 observers were compared to assess the interobserver variability for all cases.

### 2.4 Statistical analyses

The gradient (a) and correlation coefficient (r) were calculated by a single regression analysis of all data with the difference in CT values as the x-axis and the difference between the preoperative and postoperative aneurysm volumes as the y-axis. Interobserver variability was analyzed by calculating the intraclass correlation coefficients (ICC).

## 3 Results

### 3.1 Baseline characteristics and outcomes

Among 181 EVAR patients, 28 underwent postoperative contrast-enhanced CT at the 2 defined time points. The median patient age was 75 years (range, 60–87 years). Most patients were male and had a history of smoking, hypertension, and dyslipidemia. Excluder was the most frequently used stent graft (Table 1).

**Table 1:** Baseline characteristics and operative details of 28 patients.

	28 patients
Baseline characteristics	
Age (year-old)	75 (60–87)
Male: Female	21: 7
Smoke	21
Diabetes Mellitus	3
Hypertension	21
Cardiac artery disease	9
Dyslipidemia	19
Renal dysfunction	12
Stroke	2
Operative details	
Device	
Excluder	21
Endurant	5
AFX	2
Surgical duration (min)	158 (98–365)
Blood loss (ml)	83 (0–420)
Endoleaks at discharge	
Type I	0
Type II	10
Type III	1
Type IV	3

**Figure 3:**
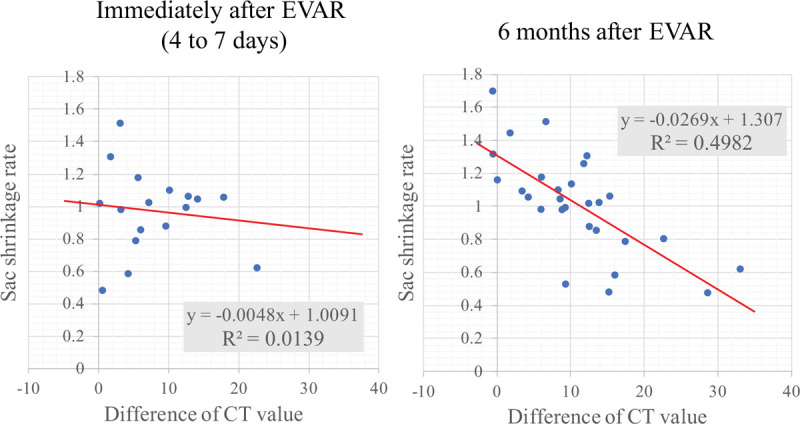
Correlation between the ratio of the AAA sac volume shrinkage rate and the difference in CT values (AWE value) immediately after (4–7 days) and 6 months after EVAR. AAA = abdominal aortic aneurysm, AWE = aneurysm wall enhancement, CT = computed tomography, EVAR = endovascular aneurysm repair.

The regression functions calculated using the AWE value immediately (4–7 days) after EVAR and the sac shrinkage rate were y = −0.0048x + 1.0091, R^2^ = 0.0139, R = −0.1179, and those using the AWE value at 6 months and the sac shrinkage rate were y = −0.0269x + 1.307, R^2^ = 0.4982, and R = −0.7059 (Fig. 3). The AWE value immediately after EVAR was correlated with the sac shrinkage rate significantly. The AWE value at 6 months after EVAR was also strongly correlated with the sac shrinkage rate.

### 3.2 Interobserver variability

The ICC of the assessment of the difference in CT values was 0.8261 (28 CT scans soon after EVAR), and the ICC of the sac shrinkage rate assessment was 0.9943 (28 CT scans during the most recent follow-up evaluation after EVAR). Therefore, we considered these measurements to be reliable.

## 4 Discussion

Aneurysm wall enhancement was originally recognized as an imaging biomarker of the risk of intracranial aneurysm rupture.^[11–13]^ Histological studies revealed that this enhancement may be subsequent to invasion of macrophages and other inflammatory cells into the aneurysm wall, which is associated with neovascularization and degeneration of the wall.^[14]^ Ito et al focused on the AWE observed on routine preoperative and postoperative EVAR CT images and predicted that a similar mechanism might cause EVAR sac enlargement after EVAR.^[10]^ However, AWE was less frequently observed in patients with aneurysm sac expansion. Considering that AWE is reportedly associated with larger AAA, higher C-reactive protein levels, thicker aneurysm walls, and more severe atheroma,^[15]^ high AWE might be a risk factor for unruptured AAA, similar to the case of intracranial aneurysms. Therefore, the mechanisms underlying increased AWE in untreated AAA and sacs after EVAR may be different. Decompression of the sac might cause neovascularization in the vasa vasorum network. This scenario is supported by a report that demonstrated that hypoperfusion of the adventitial vasa vasorum affects AAA development.^[16]^

Because the sudden halt in aortic flow after stent graft exclusion causes AAA wall remodeling, destruction, or regeneration, we consider it crucial to determine the AWE value and circumferential changes at a minimum of 2 time points. Measurements were performed immediately after and 6 months after EVAR, as reported by a previous study.^[10]^ Additionally, our AWE volumetric measurement approach utilized only 1 slice of the maximal diameter to facilitate follow-up after EVAR. This modification was not time-consuming and seemed clinically applicable.

We found a strong correlation between the AWE value at 6 months and sac shrinkage rate; however, little correlation was found with AWE immediately (4–7 days) after EVAR. These results might support a scenario in which development of neovascularization over time has a protective role that leads to sac shrinkage. Assessing the AWE at 6 months was considered as appropriate as assessing it at 1 month; however, an evaluation of the AWE at 1 week might be too early. Further studies analyzing human aortic wall samples are necessary to reveal the mechanisms underlying sac shrinkage.

A previous study reported that type 2 endoleak after EVAR is correlated with sac enlargement, that the type 2 endoleak incidence rate was approximately 18%, and that type 2 endoleak was associated with the incidence of adverse events, sac dilatation, and re-intervention.^[6]^ There is controversy regarding the threshold, timing, and strategy that should be adopted as re-intervention for sac enlargement.^[17,18]^ Prophylactic embolization of the aortic side branches is considered to be associated with less sac enlargement^[19]^; however, the procedure involves additional expenses, and its longer operative time is associated with embolization. The high AWE value, as a predictive factor for sac shrinkage, may lessen the costs and psychological burden of patients with type 2 endoleak who require frequent monitoring for potential re-intervention.

There were several limitations to this study. First, this was a retrospective study with a limited sample size, which might have caused selection bias. Second, because we assumed that the degree of decompression in the sac depends on the porosity or composition of the endovascular device used, sub-analyses based on the device should be performed. Sub-analyses might reveal device-specific effects on the aneurysm enhancement caused by the counteraction between the fabric/stent and aneurysm wall. Third, the timing of sac volume evaluation varied widely (range, 10–52 months). The timing of the evaluation should be set within a narrow range because of the tendency for drastic sac enlargement after EVAR.^[6]^ Although we selected contrast-enhanced CT at 6 months and 1 year postoperatively, and every year thereafter for the patients without renal dysfunction. For some patients with renal dysfunction, we used the modality including ultrasonography or standard CT. Such data were not included in this study, but might have resulted in the wide range of evaluation periods. Finally, only endoleak data at discharge were available. We recently stopped performing routine contrast-enhanced CT assessments of type 2 endoleaks because of the risk of contrast nephropathy. Ito et al^[10]^ hypothesized that the absence of a type 2 endoleak causes an increase in flow through the vasa vasorum. Because occult type 1 and type 3 endoleaks also cause sac enlargement, more comprehensive endoleak data of a larger population are required.

In conclusion, our volumetric analysis of the AWE value and AAA sac shrinkage revealed that high AWE values in the slice showing the maximum diameter at 6 months after EVAR were strongly associated with the sac volume shrinkage rate. High AWE values, as a predictive factor for sac shrinkage, may aid in the selection of the appropriate clinical strategy after EVAR.

## Author contributions

**Conceptualization:** Shinya Okata, Katsuyuki Hoshina.

**Data curation:** Katsuyuki Hoshina, Kazuhiro Miyahara, Takafumi Akai.

**Investigation:** Katsuyuki Hoshina, Toshio Takayama.

**Methodology:** Shinya Okata.

**Software:** Shinya Okata.

**Supervision:** Katsuyuki Hoshina, Mitsuru Matsukura.

**Validation:** Toshihiko Isaji.

**Visualization:** Kazuhiro Miyahara.

**Writing – original draft:** Shinya Okata.

**Writing – review & editing:** Katsuyuki Hoshina.

## Abbreviations

Abbreviations: AAA = abdominal aortic aneurysm, AWE = aneurysm wall enhancement, CT = computed tomography, EVAR = endovascular aneurysm repair, HU = Hounsfield units, ICC = intraclass correlation coefficient, ROI = region of interest.
